# Association between log odds of positive lymph nodes and survival in surgically treated cervical cancer patients: a SEER database–based cohort study

**DOI:** 10.1186/s12957-026-04353-z

**Published:** 2026-04-18

**Authors:** Lu Zhang, Jinjia Yang, Ping Zhang, Yuhong Shang

**Affiliations:** 1 Department of Obstetrics and Gynecology, The First Affiliated Hospital of Dalian Medical University, Dalian, Liaoning, 116011 China; 2 Department of Neurosurgery, Shengjing Hospital of China Medical University, Shenyang, Liaoning, 110004 China

**Keywords:** Cervical cancer, Nomogram, SEER, Survival, Surgery

## Abstract

**Background:**

Conventional nodal staging (N stage) and positive lymph node ratio exhibit limitations in accurately evaluating lymph node status. The current research aims to explore the prognostic value of the logarithmic odds of positive lymph nodes (LODDS) for projecting overall survival (OS) and cancer-specific survival (CSS) in patients with cervical cancer (CC) undergoing surgery.

**Methods:**

Patients who received surgical treatment for CC between 2004 and 2020 were identified from the Surveillance, Epidemiology, and End Results database. Propensity score matching (PSM) was applied to balance baseline characteristics. Hazard ratios (HRs) were analyzed employing Cox proportional hazards regression models. The possible nonlinear relationship was evaluated via restricted cubic splines. Survival rates were compared utilizing Kaplan-Meier curves. Subgroup analyses were also conducted. A novel LODDS-based model was established and verified. Its predictive accuracy was compared with that of the conventional Tumor-Node-Metastasis (TNM) staging model.

**Results:**

A total of 9,501 subjects were initially enrolled. Following PSM, 2,092 matched cases were encompassed in the analysis cohort. Multivariable analysis indicated that High LODDS was markedly related to elevated risk of mortality (OS: HR 2.04, 95% confidence interval [CI] 1.747–2.383; CSS: HR 1.821, 95% CI 1.52–2.181; *P* < 0.001). Model comparison indicated superior predictive performance for the novel LODDS-based model over the conventional model incorporating TNM staging (C-index: 0.743 vs. 0.692). Both the net reclassification improvement and integrated discrimination improvement indices confirmed that the new model offered notably better predictive capability for survival at 1, 3, and 5 years (*P* < 0.001 for all).

**Conclusion:**

LODDS constitutes an independent prognostic factor for individuals with CC following surgery. Compared with conventional TNM staging, LODDS provides incremental prognostic value, facilitating the development of personalized risk assessment and more accurate follow-up strategies.

**Supplementary Information:**

The online version contains supplementary material available at 10.1186/s12957-026-04353-z.

## Background

Cervical cancer (CC) ranks as the fourth most common malignancy among women worldwide, with approximately 600,000 new cases and 340,000 deaths annually [1]. Screening programs and widespread human papillomavirus vaccination have reduced the incidence and mortality of CC in developed nations, but its burden in developing countries remains high [1]. Furthermore, survival rates for patients diagnosed at advanced stages are still low [2].

The most frequently used scheme for staging CC is the International Federation of Gynecology and Obstetrics (FIGO) staging system. However, individual patient factors—such as race, age, histological type, treatment modality, and lymph node status—also influence patient prognosis [3–5]. Lymph node metastasis constitutes one of the most critical risk factors for postoperative recurrence and mortality in CC [6, 7]. The updated 2018 FIGO staging system incorporated nodal status: stage IIIC1 indicates pelvic lymph node metastasis, while stage IIIC2 denotes para-aortic lymph node involvement. Despite the established prognostic significance of nodal metastasis, the optimal method for its evaluation in CC remains debated. Current staging systems rely merely on the anatomic location of metastatic nodes, neglecting other aspects of nodal status.

Traditional metrics like the lymph node ratio and the number of positive lymph nodes (PLNs) are susceptible to the extent and total count of lymph node dissection. In contrast, the logarithmic odds of positive lymph nodes (LODDS) integrates information from both negative lymph node and PLN counts within the total examined lymph nodes (ELN). This logarithmically transformed indicator, theoretically, offers superior discriminative power, particularly in extreme scenarios where PLN counts are zero or equal to ELN. This metric has garnered increasing attention in recent years. LODDS has been explored in colorectal [8] and gastric cancers [9], yet studies in CC remain limited, with a particular paucity of in-depth comparisons against the conventional Tumor-Node-Metastasis (TNM) staging framework. Leveraging the Surveillance, Epidemiology, and End Results (SEER) database, our investigation employed propensity score matching (PSM), restricted cubic splines (RCS), and multiple model comparison metrics to evaluate the prognostic utility of LODDS comprehensively in individuals with CC undergoing surgery. Factors influencing patient outcomes were analyzed, and a new LODDS-based prognostic model was established to stratify individuals with CC into distinct risk categories, thereby more accurately projecting their long-term survival.

## Materials and methods

### Study population

Patient data were acquired from SEER using SEER∗Stat (v9.0.42). The study cohort comprised individuals diagnosed with primary cervical carcinoma (ICD-O-3 site: Cervix Uteri) between 2004 and 2020, confirmed by pathology and restricted to the histological subtypes of squamous cell carcinoma or cervical adenocarcinoma (Supplementary Table 1). All patients had undergone surgical treatment. We excluded individuals with incomplete records (specifically lacking information on lymph node), those who did not undergo surgery, individuals aged under 18 years, and patients whose CC was not the only primary malignancy. Approval from an ethics committee was unnecessary because the data in SEER are de-identified and publicly accessible.

### Study variables

LODDS was computed using the equation: LODDS = ln[(PLN + 0.5) / (ELN - PLN + 0.5)]. For categorization, the optimal cutoff value for dividing LODDS into distinct groups was identified by applying the maximally selected rank statistics method. Two primary endpoints were cervical cancer-specific survival (CSS) and overall survival (OS). From SEER, we collected the following information: age, marital status, diagnosis year, primary site, race, histology, tumor size, TNM stage, treatment modalities (surgery, chemotherapy, radiotherapy), and counts of negative lymph nodes and PLNs.

### Statistical analysis

Several analyses were implemented following data collection. Inter-group differences were compared via the chi-square test. To mitigate potential confounding, PSM with a 1:1 ratio and a caliper width of 0.2 was applied. Competing risk regression analyses, alongside univariable and multivariable Cox proportional hazards regression models, were applied to detect risk factors affecting OS and CSS. The potential nonlinear link between continuous LODDS values and mortality risk was examined using RCS with four knots. Survival curves were generated via the Kaplan-Meier (K-M) method, and group comparisons were conducted via the log-rank test. Models were established and compared: a conventional model based on TNM staging alone and a novel model incorporating LODDS and other clinical variables. The latter was visualized as a nomogram. The predictive capabilities of both models were compared using the concordance index (C-index). Their clinical predictive efficacy was assessed via receiver operating characteristic (ROC) curves. Their predictive accuracy was evaluated by computing the net reclassification improvement (NRI) and integrated discrimination improvement (IDI) indices. A rigorous method of internal validation based on Bootstrap (1,000 iterations) was employed to mitigate potential model overfitting, and the optimism-corrected C-index was computed.

All statistical analyses were executed in R (v4.4.2). A P-value below 0.05 suggested statistical significance.

## Results

### Baseline characteristics

**Fig. 1:**
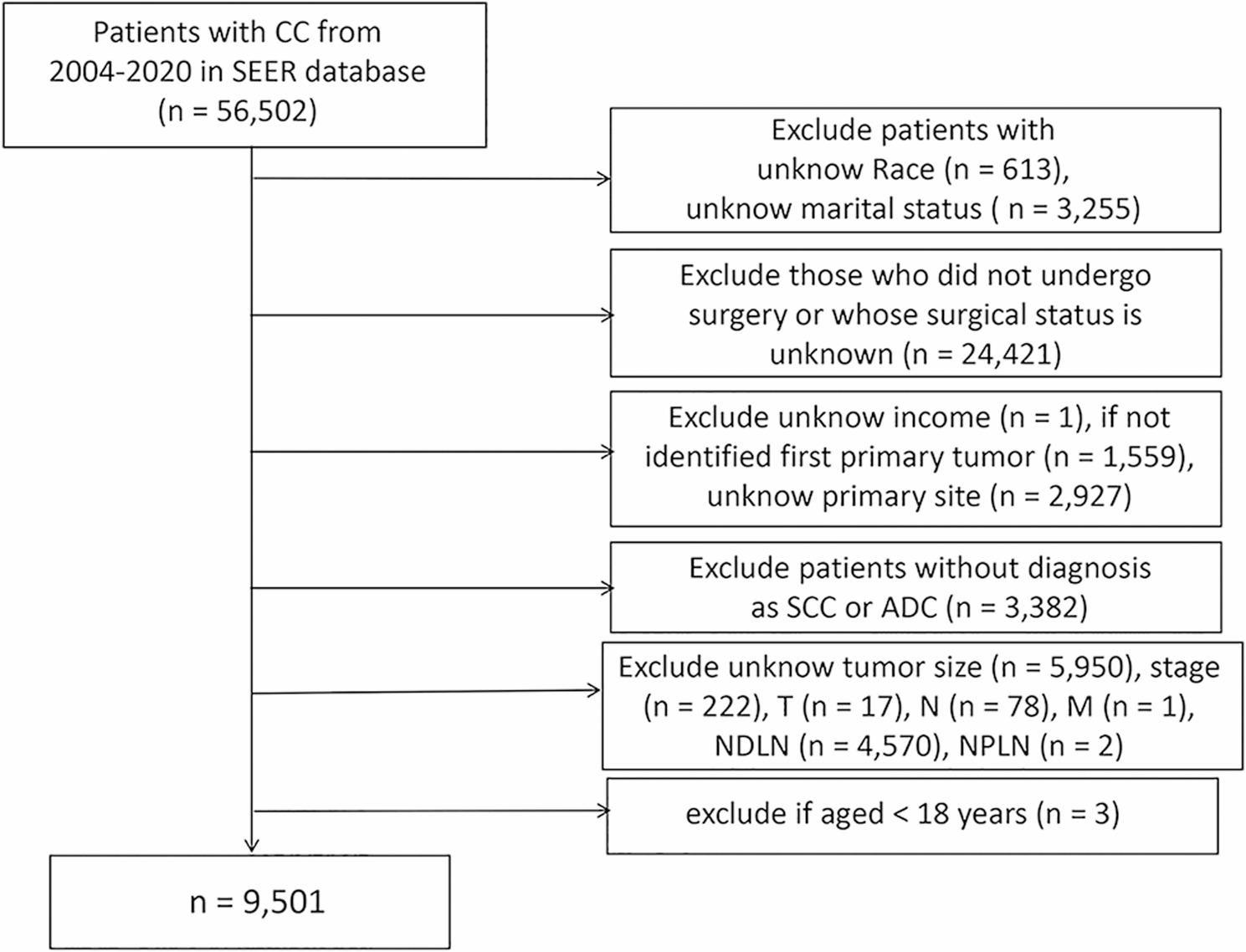
Flowchart of patient selection

From the SEER database (2004–2020), 56,502 subjects diagnosed with CC were initially identified, and 9,501 were finally included after applying the eligibility criteria (Fig. 1). The optimal cutoff of LODDS was − 0.845. Consequently, 8,267 subjects were classified into the Low-LODDS group (≤ -0.845) and 1,234 into the High-LODDS group (> -0.845). Baseline characteristics of both groups are illustrated in Supplementary Table 2. During follow-up, 1,552 all-cause deaths and 1,125 cancer-specific deaths were recorded. After PSM to control for confounders, the baseline characteristics of the two groups are presented in Table 1 (*P* > 0.05). No marked differences were noted in age, diagnosis year, race, marital status, income, histology, or receipt of surgery, chemotherapy, radiotherapy, and N stage.

**Table 1:** Baseline characteristics after propensity score matching (PSM)

Characteristic	Overall	LODDS≤-0.845	LODDS>-0.845	*p*-value^2^
	*N* = 2,092^*1*^	*N* = 1,046^*1*^	*N* = 1,046^*1*^	
Age				0.644
18–40	734(35.09%)	373(35.66%)	361(34.51%)	
40–55	854(40.82%)	430(41.11%)	424(40.54%)	
≥ 55	504(24.09%)	243(23.23%)	261(24.95%)	
Year				0.274
2004–2012	1,049(50.14%)	512(48.95%)	537(51.34%)	
2013–2020	1,043(49.86%)	534(51.05%)	509(48.66%)	
Race				0.805
Black	174(8.32%)	83(7.93%)	91(8.70%)	
Other	290(13.86%)	147(14.05%)	143(13.67%)	
White	1,628(77.82%)	816(78.01%)	812(77.63%)	
Maritalstatus				0.662
Married	1,024(48.95%)	517(49.43%)	507(48.47%)	
Unmarried	1,068(51.05%)	529(50.57%)	539(51.53%)	
Income				0.246
$70,000-$89,999	961(45.94%)	473(45.22%)	488(46.65%)	
<$69,999	509(24.33%)	245(23.42%)	264(25.24%)	
≥ 90,000	622(29.73%)	328(31.36%)	294(28.11%)	
Histology				0.318
ADC	540(25.81%)	260(24.86%)	280(26.77%)	
SCC	1,552(74.19%)	786(75.14%)	766(73.23%)	
Surgery				0.818
conservative surgery	174(8.32%)	83(7.93%)	91(8.70%)	
radical operation	1,105(52.82%)	555(53.06%)	550(52.58%)	
Standard hysterectomy	813(38.86%)	408(39.01%)	405(38.72%)	
Radiation				0.583
None/Unknown	543(25.96%)	266(25.43%)	277(26.48%)	
Yes	1,549(74.04%)	780(74.57%)	769(73.52%)	
Chemotherapy				0.352
No/Unknown	688(32.89%)	334(31.93%)	354(33.84%)	
Yes	1,404(67.11%)	712(68.07%)	692(66.16%)	
ELD	14.00(7.00,23.00)	20.00(14.00,28.00)	8.00(2.00,15.00)	< 0.001
PLD	1.00(0.00,2.00)	1.00(0.00,1.00)	2.00(0.00,3.00)	< 0.001
TumorSize				0.153
< 40 mm	1,254(59.94%)	643(61.47%)	611(58.41%)	
≥ 40 mm	838(40.06%)	403(38.53%)	435(41.59%)	
Stage				0.094
1	688(32.89%)	329(31.45%)	359(34.32%)	
2	136(6.50%)	62(5.93%)	74(7.07%)	
3	1,236(59.08%)	643(61.47%)	593(56.69%)	
4	32(1.53%)	12(1.15%)	20(1.91%)	
TStage				0.006
0–1	1,428(68.26%)	751(71.80%)	677(64.72%)	
2	527(25.19%)	235(22.47%)	292(27.92%)	
3	108(5.16%)	49(4.68%)	59(5.64%)	
4	29(1.39%)	11(1.05%)	18(1.72%)	
NStage				0.961
0	563(26.91%)	281(26.86%)	282(26.96%)	
1	1,529(73.09%)	765(73.14%)	764(73.04%)	
MStage				0.003
0	1,975(94.41%)	1,003(95.89%)	972(92.93%)	
1	117(5.59%)	43(4.11%)	74(7.07%)	

### Association analyses

#### Univariable and multivariable analyses

Multivariable analyses revealed notably elevated risks in the High-LODDS group for both all-cause deaths (HR 2.04,95%CI 1.747–2.383, *P* < 0.001) and cancer-specific deaths (HR 1.82,95%CI 1.52–2.181, *P* < 0.001).

Specifically, the multivariable Cox regression model for OS confirmed that high-LODDS individuals faced a notably elevated risk for all-cause deaths relative to those with low LODDS (HR 2.04,95% 1.747–2.383, *P* < 0.001; Table 2). The competing risk model for CSS yielded consistent results showing that high LODDS was related to increased risk for cancer-specific deaths (HR 1.82, 95%CI 1.52–2.181, *P* < 0.001; Table 3). After adjusting for confounders, LODDS remained an independent prognostic factor.

**Table 2:** Univariable and multivariable Cox regression analysis for overall survival (OS)

Characteristic	Univariable analysis		Multivariable analysis	
	HR(95%CI)	*P* value	HR(95%CI)	*P* value
LODDS				
LODDS≤-0.845	Reference		Reference	
LODDS>-0.845	2.64(2.28–3.057)	< 0.001	2.04(1.747–2.383)	< 0.001
Age at diagnosis(year)				
18–40	Reference		Reference	
40–55	1.448(1.181–1.776)	< 0.001	1.423(1.157–1.751)	< 0.001
≥ 55	2.32(1.881–2.862)	< 0.001	1.95(1.564–2.433)	< 0.001
Year at diagnosis				
2004–2012	Reference		Reference	
2013–2020	0.711(0.596–0.847)	< 0.001	0.864(0.713–1.048)	0.138
Race				
Black	Reference		Reference	
White	0.668(0.52–0.858)	0.002	0.692(0.532-0.9)	0.006
Other	0.673(0.489–0.927)	0.015	0.62(0.443–0.87)	0.006
Marital status at diagnosis				
Married	Reference		Reference	
Unmarried	1.293(1.1–1.52)	0.002	1.143(0.968–1.35)	0.115
Median household income				
$70,000–89,999	Reference		Reference	
$<69,999	1.106(0.914–1.338)	0.3	1.204(0.99–1.466)	0.063
≥$90,000	0.762(0.624–0.932)	0.008	0.98(0.798–1.204)	0.85
Histology				
ADC	Reference		Reference	
SCC	1.1(0.91–1.328)	0.325	1.031(0.849–1.253)	0.757
Tumor size				
< 40 mm	Reference		Reference	
≥ 40 mm	2.626(2.229–3.093)	< 0.001	1.881(1.576–2.246)	< 0.001
Surgery				
Conservative surgery	Reference		Reference	
Radical operation	0.817(0.612–1.091)	0.171	0.773(0.574–1.042)	0.091
Standard hysterectomy	0.881(0.656–1.184)	0.4	0.773(0.569–1.05)	0.099
Radiotherapy				
None/unknown	Reference		Reference	
Yes	1.237(1.012–1.511)	0.038	0.831(0.643–1.073)	0.156
Chemotherapy				
None/unknown	Reference		Reference	
Yes	1.284(1.071–1.54)	0.007	0.922(0.734–1.158)	0.484
Stage				
Stage 1	Reference		Reference	
Stage 2	1.474(1.01–2.151)	0.044	1.711(1.068–2.744)	0.026
Stage 3	1.48(1.213–1.805)	< 0.001	1.991(1.35–2.938)	< 0.001
Stage 4	4.365(2.554–7.462)	< 0.001	0.771(0.417–1.425)	0.407
T classification				
T0-1	Reference		Reference	
T2	2.156(1.808–2.57)	< 0.001	1.285(1.048–1.576)	0.016
T3	3.995(3.013–5.192)	< 0.001	1.9(1.406–2.569)	< 0.001
T4	7.469(4.791–11.644)	< 0.001	5.827(3.485–9.743)	< 0.001
N classification				
N0	Reference		Reference	
N1	2.113(1.679–2.659)	< 0.001	1.047(0.741–1.48)	0.793
M classification				
M0	Reference		Reference	
M1	3.51(2.75–4.481)	< 0.001	3.369(2.158–5.26)	< 0.001
ELN	NA	NA	1.001(0.993–1.01)	0.744
PLN	NA	NA	1.043(1.013–1.073)	0.004

**Table 3:** Univariable and multivariable competing risk analysis for cancer-specific survival (CSS)

Characteristic	Univariable analysis		Multivariable analysis	
	HR(95%CI)	*P* value	HR(95%CI)	*P* value
LODDS				
LODDS≤-0.845	Reference		Reference	
LODDS>-0.845	2.52(2.124–2.991)	< 0.001	1.821(1.52–2.181)	< 0.001
Age at diagnosis(year)				
18–40	Reference		Reference	
40–55	1.406( 1.133–1.744)	0.002	1.322(1.057–1.654)	0.014
≥ 55	1.678( 1.329–2.118)	< 0.001	1.315(1.017–1.699)	0.037
Year at diagnosis				
2004–2012	Reference		Reference	
2013–2020	0.667( 0.553–0.805)	< 0.001	0.844(0.682–1.043)	0.12
Race				
Black	Reference		Reference	
White	0.717(0.541–0.95)	0.02	0.68(0.5-0.926)	0.014
Other	0.738(0.518–1.05)	0.092	0.643(0.438–0.945)	0.025
Marital status at diagnosis				
Married	Reference		Reference	
Unmarried	1.117(0.936–1.331)	0.22	1.015(0.845–1.221)	0.87
Median household income				
$70,000–89,999	Reference		Reference	
$<69,999	0.993(0.804–1.226)	0.95	1.093(0.874–1.368)	0.44
≥$90,000	0.742(0.596–0.924)	0.008	0.922(0.733–1.159)	0.49
Histology				
ADC	Reference		Reference	
SCC	1.056(0.862–1.292)	0.6	0.987(0.796–1.223)	0.9
Tumor size				
< 40 mm	Reference		Reference	
≥ 40 mm	2.966(2.473–3.556)	< 0.001	2.059(1.683–2.52)	< 0.001
Surgery				
Conservative surgery	Reference		Reference	
Radical operation	0.927(0.671–1.28)	0.65	0.995(0.699–1.417)	0.98
Standard hysterectomy	0.958(0.688–1.334)	0.8	1.001(0.697–1.437)	1
Radiotherapy				
None/unknown	Reference		Reference	
Yes	1.341(1.071–1.681)	0.011	0.711(0.522–0.967)	0.03
Chemotherapy				
None/unknown	Reference		Reference	
Yes	1.602(1.299–1.975)	< 0.001	1.223(0.928–1.61)	0.15
Stage				
Stage 1	Reference		Reference	
Stage 2	1.199(0.766–1.878)	0.43	1.213(0.683–2.154)	0.51
Stage 3	1.591(1.278–1.98)	< 0.001	1.894(1.183–3.032)	0.008
Stage 4	4.565(2.561–8.137)	< 0.001	0.652(0.297–1.43)	0.29
T classification				
T0-1	Reference		Reference	
T2	2.396(1.979–2.902)	< 0.001	1.531(1.22–1.92)	< 0.001
T3	4.18(3.097–5.641)	< 0.001	1.99(1.406–2.816)	< 0.001
T4	8.337(5.043–13.782)	< 0.001	5.791(3.171–10.575)	< 0.001
N classification				
N0	Reference		Reference	
N1	2.654(2.023–3.482)	< 0.001	1.244(0.808–1.916)	0.32
M classification				
M0	Reference		Reference	
M1	3.783(2.914–4.912)	< 0.001	3.407(1.991–5.83)	< 0.001
ELN	NA	NA	1.004(0.995–1.014)	0.4
PLN	NA	NA	1.041(1.01–1.073)	0.01

#### RCS curves

**Fig. 2:**
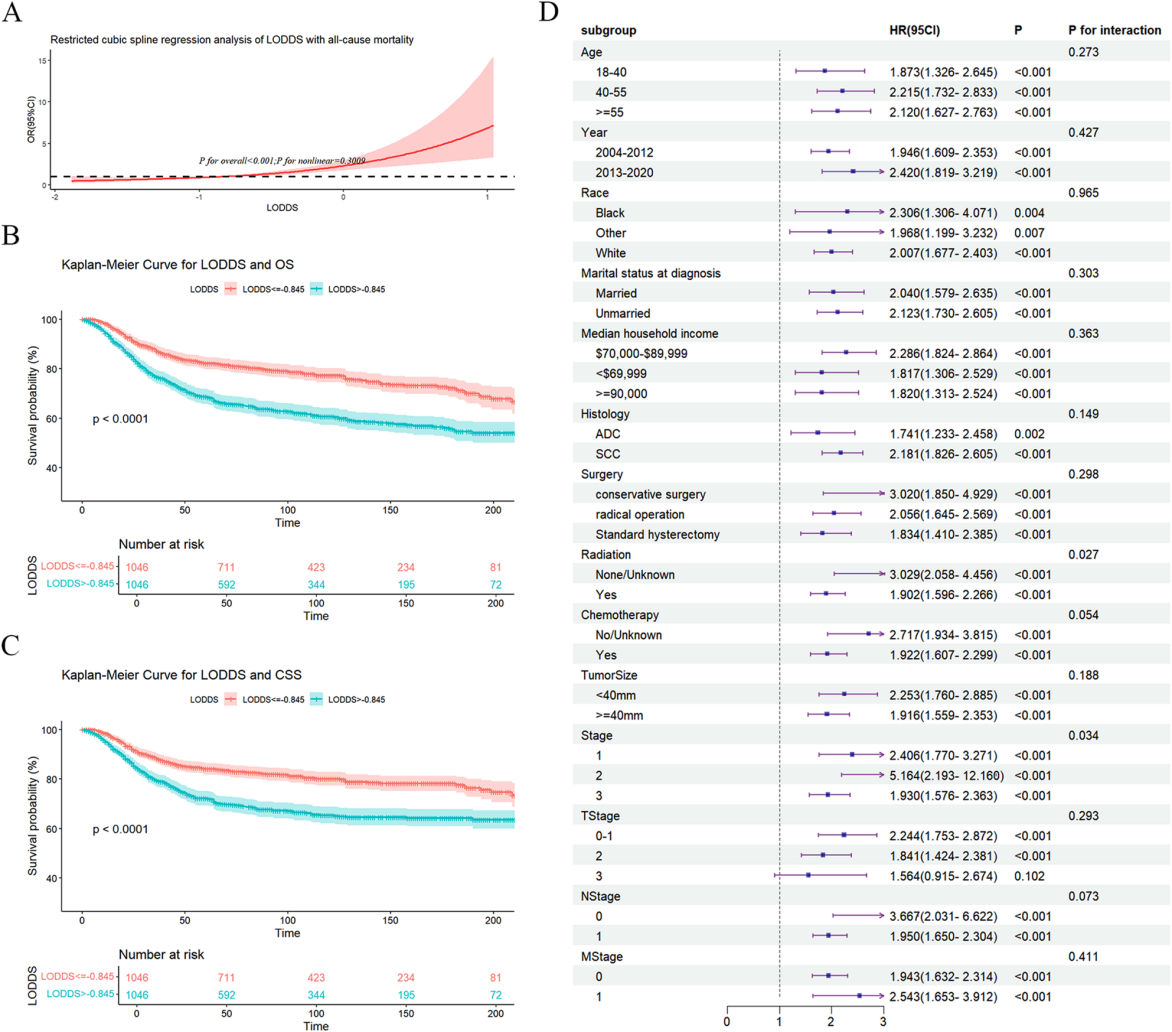
Restricted cubic spline (RCS) curves, Kaplan-Meier (K-M) survival curves, and forest plot. **A** RCS curves. These illustrate a continuous dose-response relationship between LODDS and the risk of death: as LODDS increases, the risk of death rises proportionally and continuously, with no plateau or natural turning point. **B** K-M curve for overall survival (OS) between High- and Low- LODDS groups. K-M curves show that the trajectories of survival between the two groups began to diverge clearly early after the initiation of follow-up and remained consistently separated over time; **C** K-M curve for cancer-specific survival (CSS) between High- and Low- LODDS groups; **D** Forest plot of subgroup analyses

RCS curve analysis revealed a linear link between LODDS and OS (*P* = 0.3009; Fig. 2A). This indicated a continuous dose–response relationship between LODDS and the risk of mortality: as LODDS increased, the risk of death increased proportionally without any plateau or turning point.

#### Survival analysis

The 5-year OS rate was 82.2% (95% CI: 79.8–84.6) for the Low-LODDS group, compared to 68.7% (95% CI: 65.8–71.7) for the High-LODDS group. The 10-year OS rates were 77.3% (95% CI: 74.5–80.2) and 60.6% (95% CI: 57.3–64.1), respectively. The OS-based analysis revealed that the Low-LODDS group exhibited a notably higher 5-year OS (*P* < 0.001, Fig. 2B). The CSS-based analysis indicated that the Low-LODDS group had markedly better 5-year CSS (*P* < 0.001, Fig. 2C). The trajectories of survival between the high- and low-LODDS groups began to diverge clearly early after the initiation of follow-up and remained consistently separated over time (Fig. 2B and C).

### Subgroup analysis and interaction tests

In most subgroups, the correlation of evaluated factors with outcomes was consistent, with HRs exceeding 1 and p-values below 0.05. P for interaction was above 0.05 for most factors, suggesting no significant effect modification and indicating a relatively stable main correlation across these subgroups (Fig. 2D).

Notable interactions were detected for subgroups of radiotherapy and TNM stage (P for interaction < 0.05 for both). Specifically, individuals who did not receive radiotherapy or whose radiotherapy status was unknown exhibited a higher risk of all-cause death (HR 3.29, 95%CI 2.058–4.456) than those who did receive radiotherapy (HR 1.902, 95%CI 1.596–2.266). For TNM stages, stage II patients showed the highest risk of all-cause death (HR 5.164, 95%CI 2.193–12.16), while those with stages I (HR 2.406) and III (HR 1.93) had relatively lower risk.

### Performance comparison: new LODDS-based model vs. conventional model incorporating TNM staging

Nine variables were identified from the screening. Because the comprehensive clinical stage (Stage) was mathematically derived from the T, N, and M substages, it was excluded from the modeling to avoid multicollinearity. Tumor size, radiotherapy, LODDS, T stage, N stage, M stage, chemotherapy, and surgery were incorporated in the new model (Supplementary Figs. 1 A, 1B).

**Fig. 3:**
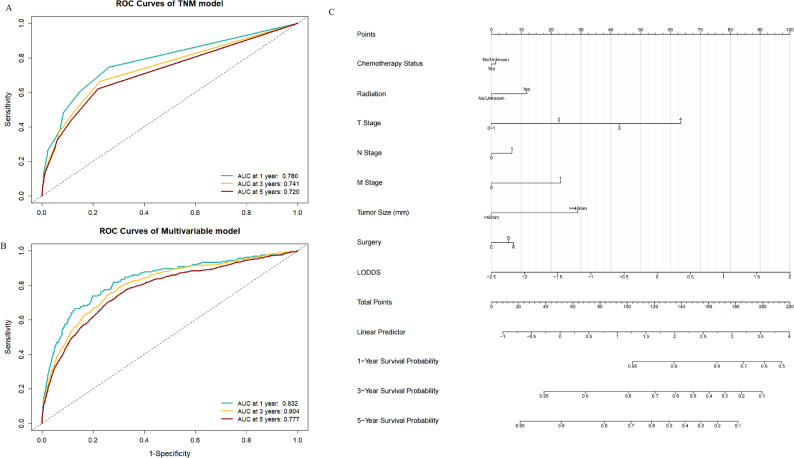
Model performance comparison and Nomogram. **A** ROC AUC for the traditional TNM staging model at 1, 3, and 5 years; **B** ROC AUC for the new predictive model incorporating LODDS at 1, 3, and 5 years; **C** Nomogram created using the LODDS-incorporated model.“Standard hysterectomy”] <- “S”. “radical operation”] <- “R”. “conservative surgery”] <- “C”

The performance of the new model was compared with that of the conventional model. The C-index was 0.692 for the conventional model and 0.743 for the new model. The C-index was 0.636 for the pN-based model, 0.638 for the LNR-based model, and 0.649 for the LODDS-based model. The areas under the ROC curves (ROC AUCs) for 1-, 3-, and 5-year OS were 0.78, 0.741, and 0.72 for the conventional model (Fig. 3A), compared to 0.832, 0.804, and 0.777 for the new model (Fig. 3B). Furthermore, accuracy improvement analysis revealed that compared to the conventional model, the new model exhibited an IDI of 0.014, 0.027, and 0.029 and an NRI of 0.352, 0.328, and 0.298 at 1, 3, and 5 years, respectively. All IDI and NRI values were above 0, with *P* < 0.001 (Supplementary Table 3). A prognostic nomogram incorporating LODDS was created (Fig. 3C). These results collectively indicate that the LODDS-based model provided incremental prognostic value relative to the conventional TNM staging system.

The apparent C-index of the multivariable model incorporating LODDS was 0.743. After Bootstrap correction, the optimism-corrected C-index remained robust at 0.741, with an extremely low optimism of 0.0014. This slight shrinkage indicated that the model was stable and the risk of severe overfitting was low.

## Discussion

Accurate categorization of lymph node involvement is paramount for staging and prognostic evaluation in CC. The present research employed PSM, RCS, and multiple model comparison metrics to thoroughly evaluate the prognostic utility of LODDS in surgically treated individuals with CC. Our findings confirm that LODDS functions as an independent predictor in this population. We also established a novel prognostic model comprising LODDS and eight other clinical variables. This composite model showed notably higher predictive accuracy than the traditional TNM staging system. The LODDS-based, multi-variable prognostic tool can aid clinicians in postoperative risk stratification, thereby guiding rehabilitation strategies and the formulation of individualized follow-up plans for CC individuals.

The updated 2018 FIGO staging system incorporated lymph node involvement—assessed via imaging or pathology [10]—reflecting the critical influence of nodal status on the prognosis of CC [11]. However, both FIGO and TNM staging systems solely consider the absence or presence of lymph node metastasis. They do not account for the precise extent of nodal disease, such as the number of positive nodes or the completeness of lymphadenectomy, nor do they integrate other risk factors. This omission can result in heterogeneous outcomes even among patients within the same clinical stage [12, 13]. The lymph node ratio, defined as the ratio of PLN to ELN, can introduce bias in scenarios with no detected nodal involvement, complete nodal involvement, or extremes in the total number of lymph nodes dissected [14, 15]. To address these limitations, we extracted and analyzed lymph node data on surgically treated patients with CC from SEER, aiming to establish a more precise prognostic model.

LODDS is an emerging metric. Its logarithmic transformation and smoothing adjustment allow it to reflect metastatic burden and, indirectly, the thoroughness of lymph node dissection. Prior research has established that LODDS maintains discriminatory power even when the lymph node ratio is 0 or 1, capturing residual heterogeneity [16]. This metric has been validated as an independent prognostic indicator across multiple malignancies, including rectal [8], gastric [9], ovarian [17], bladder [18], and thyroid cancers [19]. Previous studies comparing various lymph node staging systems for CC (e.g., TNM, FIGO, PLN, LODDS) have indicated that PLN has relatively high predictive value (C-index: 0.675), as does LODDS [20–22]. The present research encompassed a broader patient population and multiple histological subtypes of CC. We established a multivariable predictive model incorporating LODDS and conducted a multi-dimensional comparison against the traditional TNM system. Our integrated model exhibited robust discriminative ability, with a C-index of 0.728. Moreover, the positive IDI and NRI values indicate that the new model aids in preventing both undertreatment of high-risk individuals misclassified as “low-risk” by conventional TNM staging and overtreatment of truly low-risk individuals, thereby optimizing adjuvant therapeutic strategies and follow-up protocols.

In multivariable Cox regression, N stage did not exhibit independent prognostic value. This non-significance was attributable to the inclusion of LODDS. Both N stage and LODDS reflect the burden of lymph nodes; when these two highly correlated variables are placed simultaneously in a multivariable model, the competition for predictive weight is caused by multicollinearity. Because LODDS is a continuous and more refined variable, it absorbs the predictive value of N stage, rendering N stage statistically non-significant. This finding also demonstrates that LODDS surpasses the conventional N stage in predictive accuracy. Conventional N stage focuses only on the presence or absence of lymph node metastasis, disregarding the total number of nodes examined. In contrast, LODDS integrates both positive and negative lymph nodes. The K-M curves in Fig. 2B and C show that patients in the high-LODDS group experience a rapid decline in survival curves early in the follow-up period. This indicates that the trajectories of survival between the high- and low-LODDS groups began to diverge clearly early after the initiation of follow-up and remained consistently separated over time. Therefore, for patients with a high score of LODDS, clinicians may consider shortening the intervals of follow-up and adopting more aggressive adjuvant therapy. As a continuous variable, LODDS indirectly underscores the importance for gynecologic oncologists of performing adequate pelvic/paraaortic dissection of lymph nodes during surgery.

Our final model incorporated tumor size, radiotherapy, LODDS, T stage, N stage, M stage, chemotherapy, and surgery. Tumor size contributed the most substantial predictive weight, underscoring its influence on tumor staging and biological aggressiveness. Previous studies have also demonstrated that increased tumor volume in CC notably reduces 5–year survival rates. A lesion diameter of 2 cm has been proposed as a discriminative threshold, with tumors < 2 cm considered to carry a lower risk [23].

Radiotherapy is pivotal in managing CC. For early-stage disease, definitive radiotherapy offers curative outcomes equivalent to surgery [24]. For advanced or recurrent CC, concurrent chemoradiation remains the standard treatment [25]. Previous studies have demonstrated that radiotherapy can improve the survival rate of CC individuals with nodal metastasis [26]. This may be attributed to its efficacy in controlling residual micrometastases post-surgery. Our subgroup analysis revealed that radiotherapy notably modified the correlation of LODDS with survival. Specifically, the prognostic power of LODDS was markedly higher in individuals who did not receive radiotherapy or whose radiotherapy status was unknown, compared to those who did receive radiotherapy. This may be because postoperative radiotherapy effectively controls micrometastases in individuals with risk factors. For high-LODDS individuals, radiotherapy, as an effective adjuvant treatment, may partially offset the adverse effects linked to lymph node metastasis.

Several limitations should be acknowledged. First, selection bias may have occurred during the screening of patients. In SEER, data on lymph nodes were missing for some cases. To ensure the accuracy of LODDS calculation, these cases were excluded from the analysis, which may have introduced some selection bias. Second, SEER does not capture data on the recurrence or progression of tumors. Therefore, only OS and CSS could be used as primary endpoints in the current research. This may limit the comprehensive assessment of LODDS for predicting early disease recurrence. Additionally, SEER lacks detailed information on chemotherapy regimens. Although receipt of chemotherapy was included as a covariate in the analysis, adjustment for specific chemotherapy protocols was not possible given the absence of these clinical details, representing a potential confounding factor. Finally, this research lacked an external validation cohort. Although an internal Bootstrap validation method (1,000 resamples) was adopted and the optimism-corrected C-index was calculated to minimize the risk of model overfitting, internal validation cannot fully substitute for external validation. Thus, the generalizability and external applicability of the prediction model to different populations remain to be verified. Nevertheless, the large sample size and population-based nature of SEER provided reliable statistical power for the current research and support the prognostic value of LODDS in patients with CC. Future multicenter, prospective cohort studies are needed for further validation.

## Conclusion

We established and verified a novel prognostic model for CC incorporating LODDS. This model demonstrates high predictive efficacy, indicating that LODDS may serve as a valuable complementary tool to existing staging systems. The LODDS-based nomogram provides a practical instrument to help clinicians identify high-risk individuals who may benefit from more intensive adjuvant therapy or closer follow-up.

## Supplementary Information

Supplementary Material 1.

## Data Availability

The datasets generated during and/or analyzed during the current study are available from the SEER.

## Abbreviations

CC: Cervical cancer
CI: Confidence interval
C-index: Concordance index
CSS: Cervical cancer-specific survival
ELN: Examined lymph nodes
FIGO: International federation of gynecology and obstetrics
HR: Hazard ratio
IDI: Integrated discrimination improvement
K-M: Kaplan-Meier
LODDs: Logarithmic odds of positive lymph nodes
NRI: Net reclassification improvement
OS: Overall survival
PLNs: Positive lymph nodes
PSM: Propensity score matching
RCS: Restricted cubic splines
ROC: Receiver operating characteristic
SEER: Surveillance, epidemiology, and end results
TNM: Tumor-node-metastasis
