# Supplementary material for: Association between log odds of positive lymph nodes and survival in surgically treated cervical cancer patients: a SEER database–based cohort study
Source: World J Surg Oncol. 2026 Apr 18;24:237. doi: 10.1186/s12957-026-04353-z (PMC13224585; doi:10.1186/s12957-026-04353-z)
Supplement: Supplementary file 1 — Supplementary Material 1. [file 12957_2026_4353_MOESM1_ESM.docx]

**Supplementary Table 1.** Histology codes.

| **Histological subtypes** | **Histology codes** |
| --- | --- |
| Squamous cell carcinoma (SCC) | 8070,8072,8071,8076,8052,8083,8073,8074,8082,8084,8050,8075,8078,8085 |
| Cervical adenocarcinoma (ADC) | 8140,8260,8262,8144,8384,8380 |

**Supplementary Table 2.** Original baseline characteristics.

| **Characteristic** | **Overall** | **LODDS≤-0.845** | **LODDS>-0.845** | **p-value***^2^* |
| --- | --- | --- | --- | --- |
|  | **N = 9,501*^1^*** | **N = 8,267*^1^*** | **N = 1,234*^1^*** |  |
| Age |  |  |  | <0.001 |
| 18-40 | 3,561(37.48%) | 3,149(38.09%) | 412(33.39%) |  |
| 40-55 | 3,856(40.59%) | 3,360(40.64%) | 496(40.19%) |  |
| ≥55 | 2,084(21.93%) | 1,758(21.27%) | 326(26.42%) |  |
| Year |  |  |  | 0.185 |
| 2004-2012 | 4,933(51.92%) | 4,314(52.18%) | 619(50.16%) |  |
| 2013-2020 | 4,568(48.08%) | 3,953(47.82%) | 615(49.84%) |  |
| Race |  |  |  | 0.003 |
| Black | 739(7.78%) | 619(7.49%) | 120(9.72%) |  |
| Other | 1,201(12.64%) | 1,026(12.41%) | 175(14.18%) |  |
| White | 7,561(79.58%) | 6,622(80.10%) | 939(76.09%) |  |
| Maritalstatus |  |  |  | 0.006 |
| Married | 4,969(52.30%) | 4,369(52.85%) | 600(48.62%) |  |
| Unmarried | 4,532(47.70%) | 3,898(47.15%) | 634(51.38%) |  |
| Income |  |  |  | 0.012 |
| $70,000-$89,999 | 4,333(45.61%) | 3,750(45.36%) | 583(47.24%) |  |
| <$69,999 | 2,252(23.70%) | 1,936(23.42%) | 316(25.61%) |  |
| ≥90,000 | 2,916(30.69%) | 2,581(31.22%) | 335(27.15%) |  |
| Histology |  |  |  | <0.001 |
| ADC | 3,501(36.85%) | 3,162(38.25%) | 339(27.47%) |  |
| SCC | 6,000(63.15%) | 5,105(61.75%) | 895(72.53%) |  |
| Surgery |  |  |  | <0.001 |
| conservative surgery | 601(6.33%) | 464(5.61%) | 137(11.10%) |  |
| radical operation | 5,384(56.67%) | 4,757(57.54%) | 627(50.81%) |  |
| Standard hysterectomy | 3,516(37.01%) | 3,046(36.85%) | 470(38.09%) |  |
| Radiation |  |  |  | <0.001 |
| None/Unknown | 5,913(62.24%) | 5,607(67.82%) | 306(24.80%) |  |
| Yes | 3,588(37.76%) | 2,660(32.18%) | 928(75.20%) |  |
| Chemotherapy |  |  |  | <0.001 |
| No/Unknown | 6,900(72.62%) | 6,534(79.04%) | 366(29.66%) |  |
| Yes | 2,601(27.38%) | 1,733(20.96%) | 868(70.34%) |  |
| ELD | 16.00(10.00,23.00) | 17.00(11.00,24.00) | 8.00(2.00,15.00) | <0.001 |
| PLD | 0.00(0.00,0.00) | 0.00(0.00,0.00) | 2.00(1.00,4.00) | <0.001 |
| TumorSize |  |  |  | <0.001 |
| <40mm | 7,348(77.34%) | 6,667(80.65%) | 681(55.19%) |  |
| ≥40mm | 2,153(22.66%) | 1,600(19.35%) | 553(44.81%) |  |
| Stage |  |  |  | <0.001 |
| 1 | 7,317(77.01%) | 6,895(83.40%) | 422(34.20%) |  |
| 2 | 699(7.36%) | 606(7.33%) | 93(7.54%) |  |
| 3 | 1,425(15.00%) | 745(9.01%) | 680(55.11%) |  |
| 4 | 60(0.63%) | 21(0.25%) | 39(3.16%) |  |
| TStage |  |  |  | <0.001 |
| 0-1 | 8,067(84.91%) | 7,354(88.96%) | 713(57.78%) |  |
| 2 | 1,180(12.42%) | 787(9.52%) | 393(31.85%) |  |
| 3 | 201(2.12%) | 102(1.23%) | 99(8.02%) |  |
| 4 | 53(0.56%) | 24(0.29%) | 29(2.35%) |  |
| NStage |  |  |  | <0.001 |
| 0 | 7,727(81.33%) | 7,445(90.06%) | 282(22.85%) |  |
| 1 | 1,774(18.67%) | 822(9.94%) | 952(77.15%) |  |
| MStage |  |  |  | <0.001 |
| 0 | 9,279(97.66%) | 8,183(98.98%) | 1,096(88.82%) |  |
| 1 | 222(2.34%) | 84(1.02%) | 138(11.18%) |  |

**Supplementary Table 3.** Model comparison: integrated discrimination improvement (IDI) and net reclassification improvement (NRI) values.

| **Model** | **IDI(95%CI)** | **P value** | **NRI(95%CI)** | **P value** |
| --- | --- | --- | --- | --- |
| 1-year OS |  |  |  |  |
| TNM classification | Reference |  | Reference |  |
| Multivariable model | 0.014(0.006-0.024) | P<0.0001 | 0.352(0.261-0.446) | P<0.0001 |
| 3-year OS |  |  |  |  |
| TNM classification | Reference |  | Reference |  |
| Multivariable model | 0.027(0.019-0.038) | P<0.0001 | 0.328(0.272-0.369) | P<0.0001 |
| 5-year OS |  |  |  |  |
| TNM classification | Reference |  | Reference |  |
| Multivariable model | 0.029(0.021-0.038) | P<0.0001 | 0.298(0.251-0.337) | P<0.0001 |


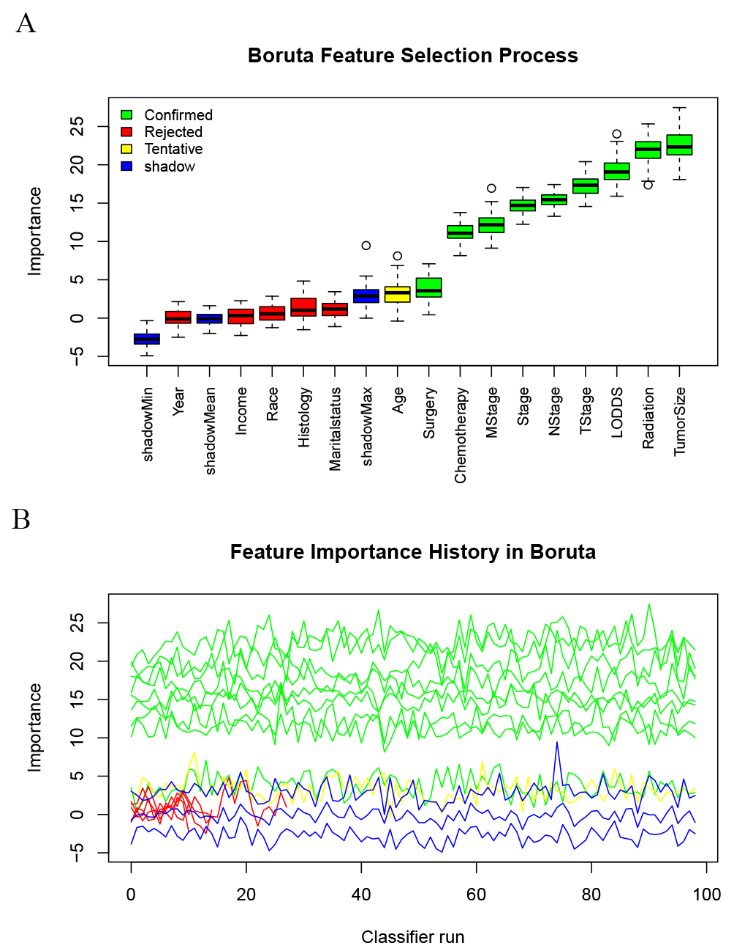


**Supplementary Figure 1** (A) Variable selection based on Burota feature importance; (B) Variable selection based on Burota feature importance.
